# Suppressor of IKKepsilon forms direct interactions with cytoskeletal proteins, tubulin and α‐actinin, linking innate immunity to the cytoskeleton

**DOI:** 10.1002/2211-5463.12454

**Published:** 2018-06-19

**Authors:** Halie A. Sonnenschein, Kenneth F. Lawrence, Karli A. Wittenberg, Frank A. Slykas, Emerald L. Dohleman, Jilan B. Knoublauch, Sean M. Fahey, Timothy M. Marshall, James D. Marion, Jessica K. Bell

**Affiliations:** ^1^ Department of Chemistry and Biochemistry University of San Diego CA USA; ^2^ Department of Immunology and Microbiology Virginia Commonwealth University Richmond VA USA; ^3^ Department of Biochemistry and Molecular Biology Virginia Commonwealth University Richmond VA USA

**Keywords:** alpha‐actinin, cytoskeleton, innate immunity, SIKE, tubulin

## Abstract

Suppressor of IKKepsilon (SIKE) is associated with the type I interferon response of the innate immune system through TANK‐binding kinase 1 (TBK1). Originally characterized as an endogenous inhibitor of TBK1 when overexpressed in viral infection and pathological cardiac hypertrophic models, a mechanistic study revealed that SIKE acts as a high‐affinity substrate of TBK1, but its function remains unknown. In this work, we report that scratch assay analysis of parental and SIKE CRISPR/Cas9 knockout HAP1 cells showed an ~ 20% decrease in cell migration. Investigation of the SIKE interaction network through affinity purification/mass spectrometry showed that SIKE formed interactions with cytoskeletal proteins. In immunofluorescence assays, endogenous SIKE localized to cytosolic puncta in both epithelial and myeloid cells and to nuclear puncta in myeloid cells, while in epithelial cells additional staining occurred in stress fiber‐like structures and adjacent to the plasma membrane. Using cellular markers, co‐occurrence of SIKE fluorescence with actin, α‐actinin, and ezrin was detected. Reciprocal immunoprecipitation revealed a SIKE:tubulin interaction sensitive to the phosphorylation state of SIKE, but a SIKE:α‐actinin interaction was unchanged by SIKE phosphorylation. *In vitro* precipitation assays confirmed a direct SIKE interaction with tubulin and α‐actinin. These results indicate that SIKE may promote cell migration by directly associating with the cytoskeleton. In this role, SIKE may mediate cytoskeletal rearrangement necessary in innate immunity, but also link a key catalytic hub, TBK1, to the cytoskeleton.

**Database:**

The mass spectrometry proteomics data have been deposited to the ProteomeXchange Consortium via the PRIDE [1] partner repository with the dataset identifier PXD007262.

**Abbreviations**
SIKE: suppressor of IKKepsilon
TBK1: TANK‐binding kinase 1

The innate immune system provides the first line of defense against invading pathogen. Pathogen detection through pathogen‐ or danger‐associated molecular patterns by innate immune receptors such as Toll‐like receptors (TLRs) initiates a myriad of responses ranging from pro‐inflammatory to type I interferon production, and apoptosis 2. Receptor signaling flows through catalytic hubs, such as kinases, that activate, for example, transcription factors to elicit the aforementioned host defenses. In addition to these well‐studied host cell signaling pathways, catalytic hubs modify other targets for which the downstream purpose within the innate immune response was initially not well understood. For example, optineurin, an autophagy receptor, was originally characterized as an inhibitor of the catalytic hub, TANK‐binding kinase 1 3, but has subsequently been identified as a TBK1 substrate 4, 5 and essential for TBK1's recruitment and activation on mitochondrion 6. Oft times, noncanonical substrates of catalytic hubs reveal a linkage between the innate immune system's detection component and the host's response to these signals.

Suppressor of IKKepsilon (SIKE) intersects with the catalytic hub, TANK‐binding kinase 1, which is associated with the activation of interferon regulatory factors (IRFs) to initiate a type I interferon response 7. Initially, SIKE was classified as an endogenous inhibitor of the TBK1‐mediated activation of IRFs downstream of the antiviral responses mediated by TLR3 and RIG‐I 8. Kinetic characterization of SIKE's inhibitory mechanism revealed that SIKE was a high‐affinity substrate of TBK1 9. Thus, when present at a sufficient concentration, SIKE is able to block TBK1's phosphorylation of its canonical substrates, IRFs 3 and 7. In cardiac tissue, cardiac hypertrophy reduced SIKE expression levels, but overexpression of SIKE protected against aortic banding‐induced or agonist‐induced cardiac hypertrophy by blocking TBK1‐mediated activation of Akt 10. In both studies, the ascribed TBK1 inhibitory role of SIKE relied upon the overexpression of SIKE to reveal a functional phenotype, but neither study addressed the role of endogenous SIKE.

Insights into potential functional relationships for SIKE arise from a high‐density interaction map surrounding the protein phosphatase 2A catalytic subunit 11. SIKE and a highly homologous protein, fibroblast growth factor receptor 1 oncoprotein 2 (FGFR1OP2), complexed to sarcolemmal membrane‐associated protein within the larger striatin‐interacting phosphatase and kinase (STRIPAK) complex. STRIPAK complexes, which vary in composition based upon function, are supramolecular complexes that utilize their kinase/phosphatase associated activities to regulate signaling, cell cycle control, apoptosis, vesicular trafficking, Golgi assembly, cell polarity, cell migration, neural and vascular development, and cardiac function 12. Although human SIKE‐containing STRIPAK complex has no attributed function, Drosophila SIKE‐containing STRIPAK complex negatively regulates the Hippo pathway that controls final tissue and organ size by inhibiting cell proliferation and promoting apoptosis 13. In this process, SIKE formed bridging interactions with the target, Hpo kinase, and Drosophila striatin in the STRIPAK complex. Another study in fly motor neurons implicated the SIKE‐containing STRIPAK complex in the regulation of synapse formation by modulating actin organization 14. These studies imply that, beyond SIKE's interaction with TBK1, SIKE may function within a larger macromolecular complex.

Based on these earlier studies, we focused on delineating a function for endogenous SIKE. Knocking out SIKE expression by a CRISPR/Cas9 approach reduced cellular migration compared to parental cells, suggesting SIKE may function in cytoskeletal rearrangement. To further characterize a SIKE : cytoskeletal interaction, we defined a SIKE interaction network using co‐immunoprecipitation of epitope‐tagged SIKE coupled to LC‐MS/MS and investigated the validity of these interactions through immunofluorescence assays (IFAs) and reciprocal immunoprecipitations (RcIPs). Here, we report that endogenous SIKE showed a cell‐type‐dependent localization. IFAs and RcIPs demonstrated that SIKE formed a complex with cytoskeletal proteins, tubulin and α‐actinin. dsRNA stimulation to induce SIKE phosphorylation or use of phosphomimetic SIKE enhanced the SIKE : tubulin interaction. To distinguish direct versus indirect interactions with SIKE, *in vitro* precipitation (IVP) reactions confirmed that the SIKE : cytoskeletal protein interactions were direct. Our findings indicated that SIKE directly associated with the cytoskeleton and that the loss of SIKE impaired cellular migration thus demonstrating its potential role in mediating cytoskeletal rearrangement necessary in innate immunity, but also its potential to link a key catalytic hub, TBK1, to the cytoskeleton.

## Results

### SIKE knockout cells show impaired cell migration

**Figure 1:**
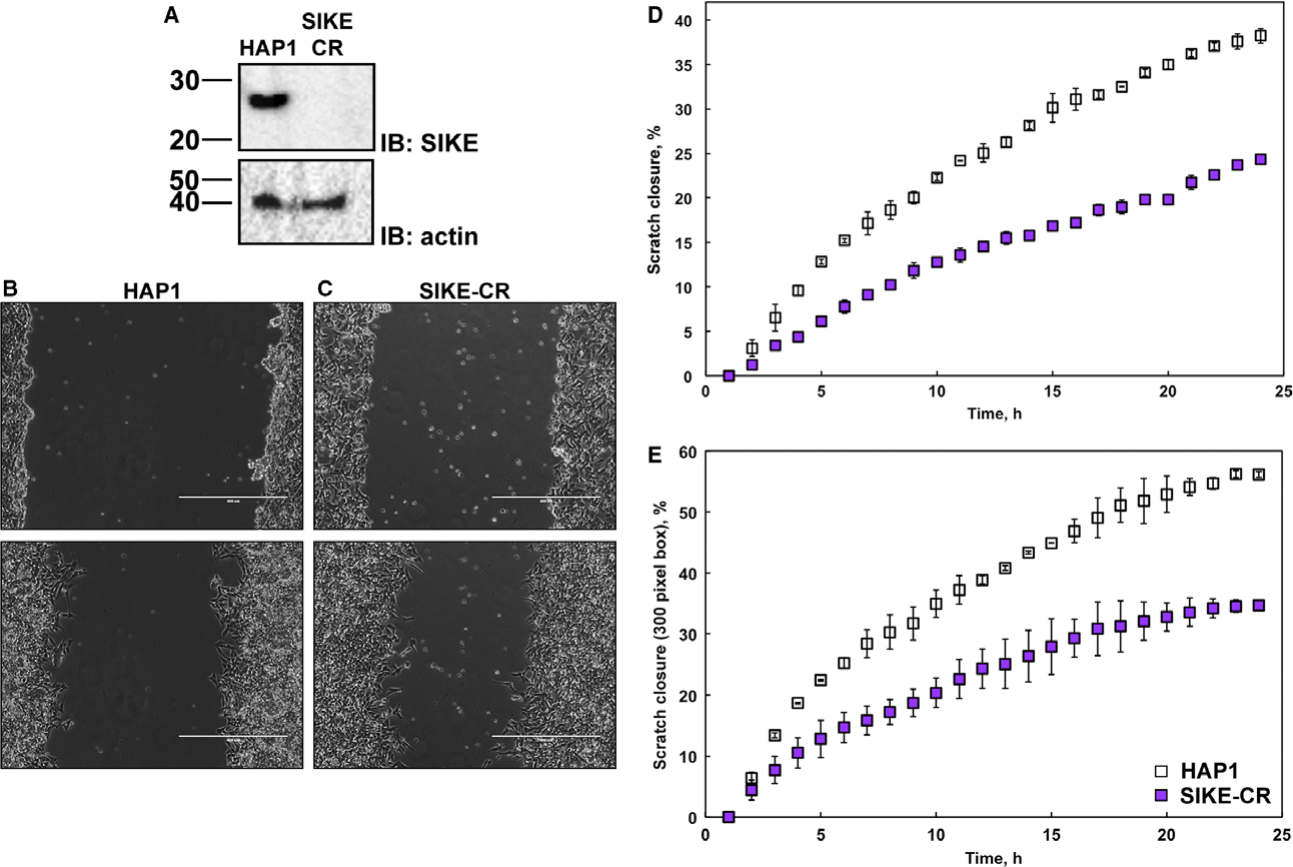
Cells lacking SIKE show decreased migration. A SIKE knockout cell line was generated using the CRISPR/Cas9 technique. (A) Immunoblot of parental, HAP1, and SIKE knockout, SIKE‐CR, lysates (20 μg protein/lane) probed with anti‐SIKE antibody confirmed that the SIKE‐CR cell lines lacked SIKE expression. Actin served as a loading control. Representative images of scratch assays of HAP1 (B) and SIKE‐CR (C) collected over a 24‐h time period to assess cell migration. Top, 0 h; bottom, 24‐h time points. Scale bar (white bar) is 400 μm. Analyses of entire scratch (D) or 300 pixels from cell/scratch boundary (E) showed that SIKE‐CR cells consistently migrated less (migration into scratch area reduced by ~ 14% (entire scratch) to ~ 21% (300 pixel analysis)) and slower into the scratch area (at 5 h, 1.5 versus 3.1 slope for SIKE‐CR and HAP1, respectively). Error represented by standard deviation of three independent experiments. Significantly different values for % closure (95% confidence interval, *P *≤* *0.05) were assessed by Student's unpaired *t*‐test, two‐tailed. Data points 1–3 and 1–2 of the full versus 300 pixel analyses, respectively, were not significantly different values by this assessment.

One defining response of innate immune defenses is cytoskeletal rearrangements to initiate cell movement either for migration or for phagocytosis. To assess if the biological role of SIKE was related to these host defense mechanisms, we examined cell migration via a scratch assay using a parental, HAP1, cell line and a HAP1 CRISPR/Cas9 SIKE knockout cell line, SIKE‐CR. Loss of SIKE expression in the SIKE‐CR cell line was confirmed via immunoblot (Fig. 1A). Confluent HAP1 or SIKE‐CR cells, plated in 35‐mm dishes with low sera medium, were scratched, and dead/loose cells were removed by PBS washing, replenished with complete medium, and imaged for 24 h (Fig. 1B,C). Data were analyzed in two ways, percentage of scratch closure (Fig. 1D) and percentage of scratch closure within 300 pixels from cell/scratch boundary edge (Fig. 1E). The second approach addressed any differences in scratch width between experiments. In both analyses, significantly decreased migration (*P *≤* *0.05) was observed for the SIKE knockout cell line, ranging from 13.9% to 21.4% for scratch closure of entire area or 300‐pixel box, respectively.

### SIKE interaction network identified by co‐immunoprecipitation tandem MS/MS

**Figure 2:**
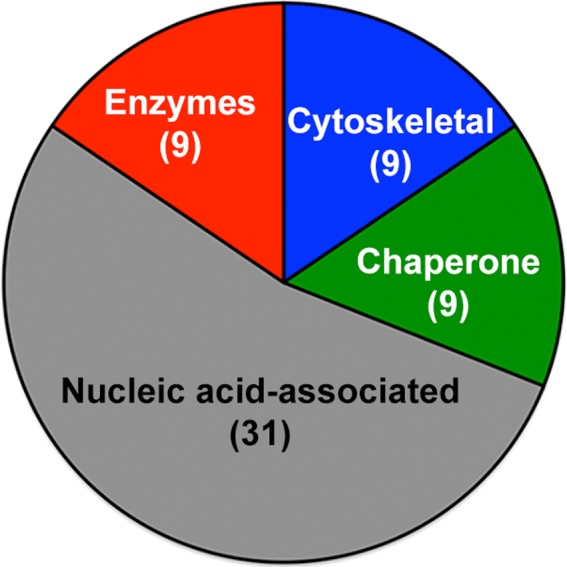
Co‐immunoprecipitation of FLAG‐tagged SIKE followed by tandem MS/MS identified several classes of SIKE interacting proteins. Using Protein Prophet algorithm for protein identification (probability > 90%), 21 protein partners for SIKE were identified and classified into four broad categories, nucleic acid binding, chaperone, enzyme, and cytoskeletal. As these binding partners are typically identified as contaminants of proteomic experiments, further validation through immunofluorescence assays and reciprocal immunoprecipitations was required.

To identify a SIKE interaction network that would provide insight into how SIKE might associate with cytoskeletal structures, we used co‐immunoprecipitation coupled to tandem MS/MS to identify a SIKE interaction network. In this assay, SIKE, epitope tagged with FLAG, was transiently expressed in HEK293 cells, an easily transfected epithelial cell line. Lysates for basal or dsRNA (polyriboinosinic : polyribocytidylic acid (pI : pC)) stimulated conditions to mimic a viral infection that would induce a dsRNA‐mediated antiviral response were assessed. Immunoprecipitated SIKE and associated proteins were subjected to tryptic digestion followed by LC‐MS/MS to identify components of the precipitated networks. SIKE interactions were categorized into four types: cytoskeletal proteins, chaperones, nucleic acid‐associated, and enzyme (Fig. 2, Table 1).

**Table 1:** SIKE interactome summary. FLAG‐tagged SIKE was immunoprecipitated from HEK293 lysates, subjected to tryptic digest and resulting peptides analyzed by LC‐MS/MS. Data were analyzed with sequest and x!tandem software

		Protein name	MW, kDa	Accession nos UniProt	WT							
					Basal				dsRNA			
					Run 1		Run 2		Run 1		Run 2	
					% cov	SC	% cov	SC	% cov	SC	% cov	SC
BAIT		Suppressor of IKKepsilon	24	Q9BRV8	71	48	82	272	71	44	82	197
PREY	Cytoskeleton	aActin, cytoplasmic 1	42	P60709			26	27			20	9
		aActin, aortic smooth muscle	42	P62736			18	15			7.2	5
		Actin, gamma 1	40	P63261	13	5			9.6	5		
		Tubulin alpha‐1C chain	58	F5H5D3			13	11			6.7	5
		bTubulin beta chain	49	P07437			28	25			14	6
		bTubulin beta‐4B chain	50	P68371			21	17			11	3
	Chaperone	cHeat shock 70‐kDa protein 1A/1B	70	P0DMV8/P0DMV9	13	7	23	44	7.5	6	19	20
		cHeat shock 70‐kDa protein 6	71	P17066			9.8	15			8.7	11
		Heat shock cognate 71‐kDa protein	54	P11142	13	5			13	6		
		Heat shock protein HSP 90‐beta	83	P08238			5.4	5			1.9	2
		Peptidyl‐prolyl cis‐trans isomerase A	18	P62937	33	5			16	1		
	DNA/RNA associated	Nucleophosmin 1, isoform 2	29	P06748‐2	5.3	2	18	12	9.4	2	18	8
		Treacle protein	152	Q13428	1.9	3			2.5	4		
		Elongation factor 1‐alpha 1	50	P68104	15	3	7.6	3	4.8	3	2.4	2
		Activated RNA polymerase II transcriptional coactivator p15	14	P53999	20	4			19	2		
		UPF0568 protein C14orf166	28	Q9Y224	18	2			5.7	2		
		Heterogeneous nuclear ribonucleoproteins A2/B1	36	P22626			21	12			13	7
		THO complex subunit 4/ALYREF	27	Q86V81	10.9	3			10.9	4		
	Enz.	Lactoylglutathione lyase	21	Q04760	21.2	2			9.2	2		
		Alpha‐enolase	47	P06733	17	3	13	8	17	6	6.9	4
		Glyceraldehyde‐3‐phosphate dehydrogenase	32	P04406			16	7			9.9	4

^a^ Within cluster of actin, WT expt. 2; ^b^ within tubulin beta chain cluster, WT expt. 2. % cov, percent coverage of MS identified sequence for protein sequence; ^c^ within cluster of heat shock 70‐kDa protein 1A/1B, WT expt. 2; SC, spectra count.

### Endogenous SIKE colocalizes with cytoskeletal proteins

**Figure 3:**
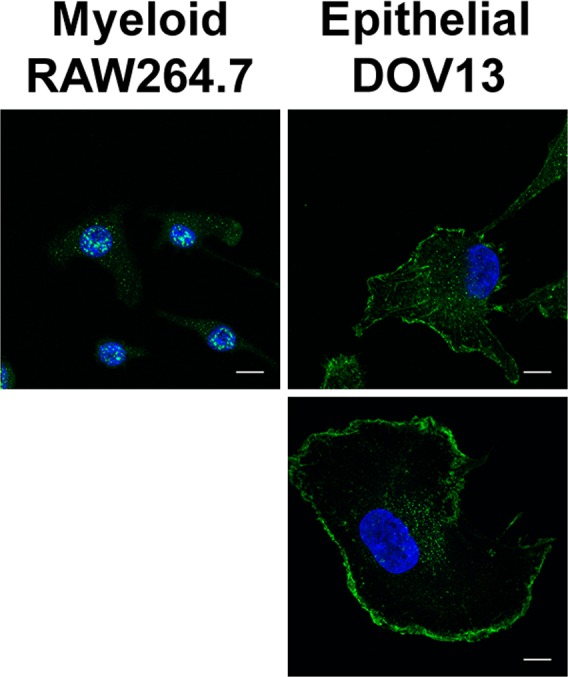
Cell‐type‐dependent localization of endogenous SIKE. Mouse macrophage RAW264.7 or human ovarian epithelial DOV13 cells were fixed, permeabilized, and stained with primary rabbit antibody for SIKE, secondary antibody for anti‐rabbit Alexa Fluor 488 (green) and Hoechst 33342 (blue) at original magnification 63×. Images are maximum intensity of *z*‐stack projections. Scale bar, 10 μm. Macrophages show nuclear and cytosolic puncta, whereas epithelial cells have only cytosolic puncta, but also show SIKE in stress fiber‐like structures as well at the plasma membrane. Images are representative of two independent experiments, five images/experiment.

To validate SIKE interactions identified by tandem MS/MS colocalization assays, immunofluorescence assays of endogenous SIKE and cellular markers were completed. Endogenous SIKE localization was examined in two cell types associated with initial host–pathogen interactions, epithelial, which line surfaces exposed to the host's environment, and myeloid, which represent immune cell lineages (Fig. 3). RAW264.7 cells, a mouse macrophage cell line, showed SIKE primarily in distinct cytosolic and nuclear puncta, whereas in DOV13 cells, a human ovarian epithelium cell line, SIKE is excluded from the nucleus and is found in cytosolic puncta, stress fiber structures, and underlying the plasma membrane. These data indicate cell‐type‐dependent and/or species‐specific localization of SIKE.

**Figure 4:**
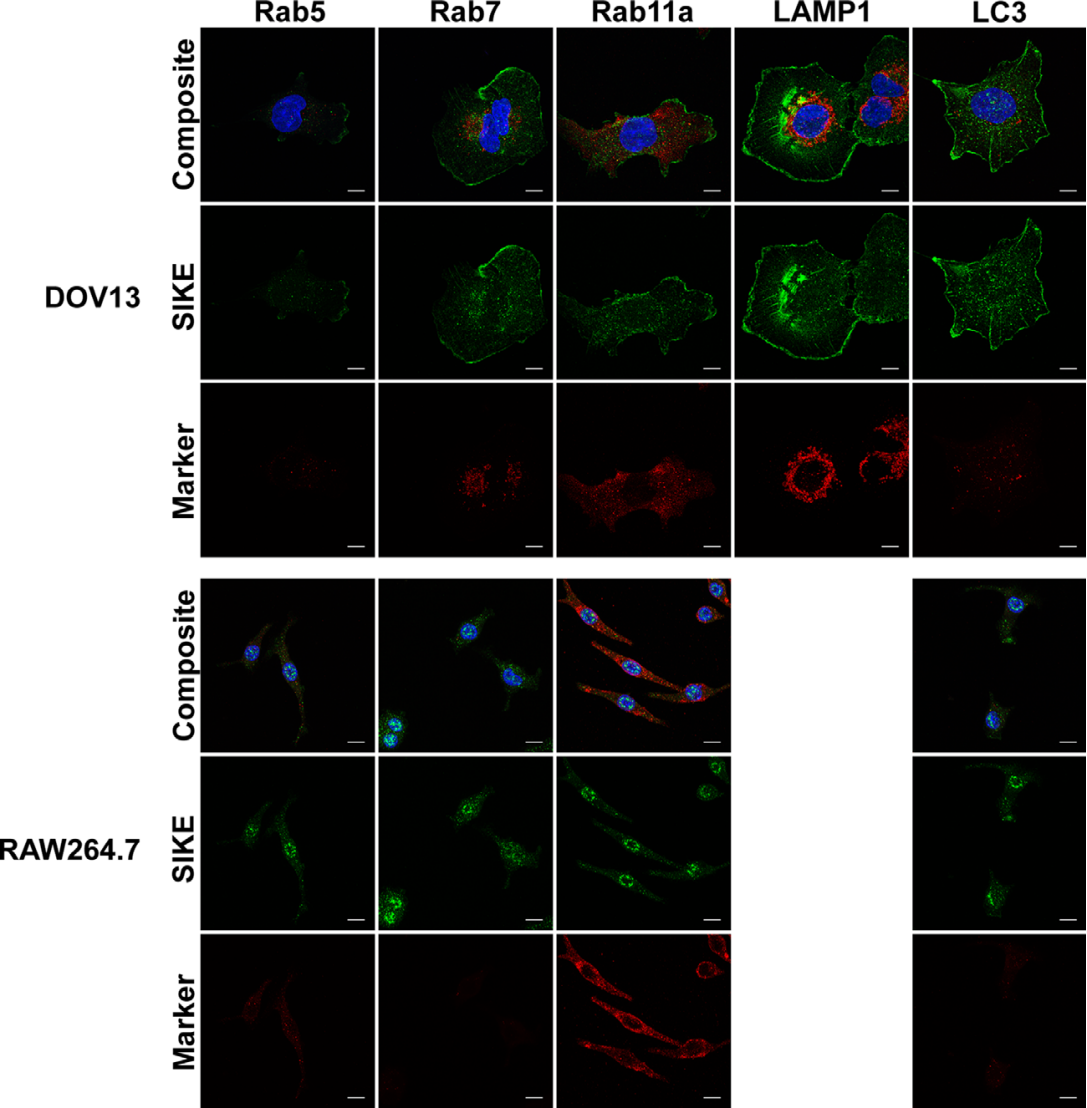
SIKE does not colocalize with classic endosomal markers. DOV13 or RAW264.7 cells were fixed, permeabilized, and stained with mouse anti‐endosomal marker antibody as indicated followed by anti‐mouse Alexa Fluor 555 (red), rabbit anti‐SIKE antibody followed by anti‐rabbit Alexa Fluor 488 (green) and Hoechst 33342 (blue). Endosomal markers: early, Rab5; late, Rab7; recycling/plasma membrane, Rab11; lysosome, LAMP1; autophagosomes, LC3. Images are maximum intensity of *z*‐stack projections. Scale bar, 10 μm. Images are representative of two independent experiments, five images/experiment. No significant overlap of the cytoskeletal and SIKE fluorescence was observed.

**Figure 5:**
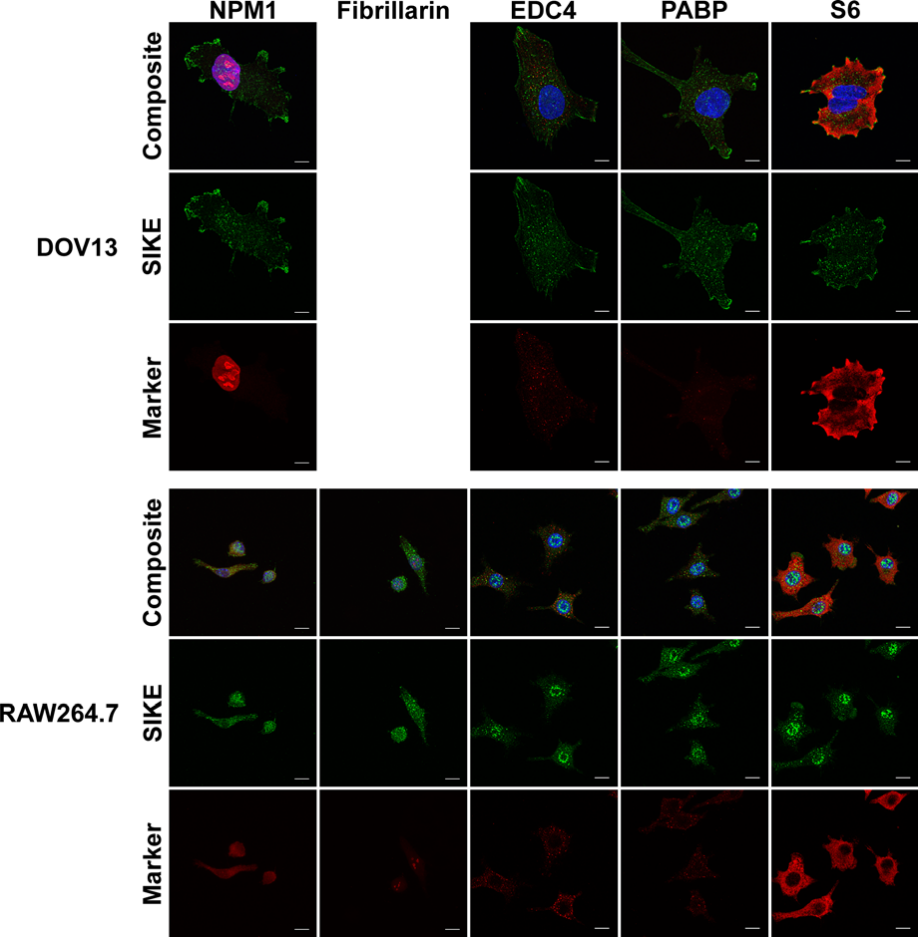
SIKE does not colocalize with nuclear, RNA granule, or ribosome markers. DOV13 or RAW264.7 cells were fixed, permeabilized, and stained with mouse anti‐nucleic acid‐associated protein marker antibody as indicated followed by anti‐mouse Alexa Fluor 555 (red), rabbit anti‐SIKE antibody followed by anti‐rabbit Alexa Fluor 488 (green) and Hoechst 33342 (blue). Nucleic acid‐associated markers: nucleophosmin 1 (NPM1), nucleolus; fibrillarin, nucleolus; enhancer of mRNA decapping 4 (EDC4), processing bodies; poly(A) binding protein 1 (PABP), stress granules; S6, ribosomal protein. Images are maximum intensity of *z*‐stack projections. Scale bar, 10 μm. Images are representative of two independent experiments, five images/experiment. No significant overlap of the nucleic acid‐associated protein and SIKE fluorescence was observed.

**Figure 6:**
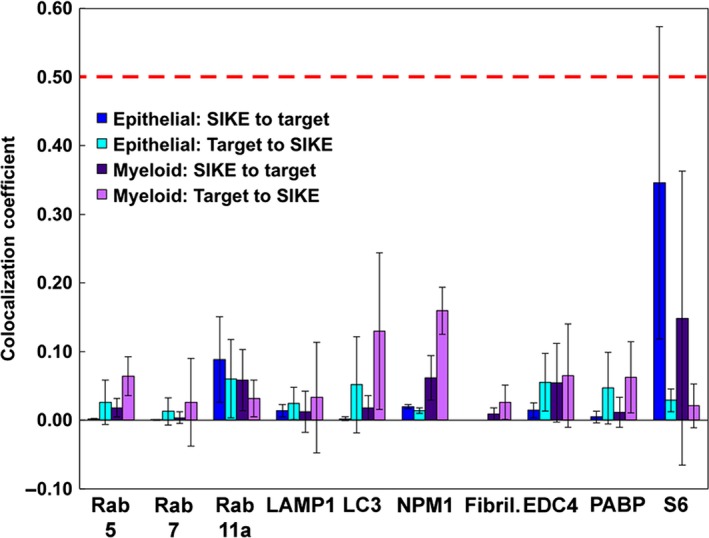
Quantitation of colocalization between SIKE and endosomal or nucleic acid‐associated proteins. zen software colocalization function (Carl Zeiss Microscopy) was employed on thresholded images to determine the unweighted colocalization coefficient for SIKE to target and target to SIKE intersection of fluorescence. Error bars represent standard deviation for *n* = 6. For all markers, the intersection of fluorescence is not significantly greater than 0.5 (indicated by red dotted line), accounting for ≤ 50% of pixel population for SIKE or marker.

To classify the structures and proteins to which SIKE was associating, colocalization assays via immunofluorescence were completed. Two analyses of SIKE fluorescence with well‐characterized cellular markers were undertaken: co‐occurrence and codistribution. Co‐occurrence using a Mander's overlap coefficient measured the overlap of fluorescence between SIKE and the cellular marker from the perspective of SIKE fluorescence (how much SIKE fluorescence overlapped with the cellular marker fluorescence?) and the cellular marker (how much cellular marker fluorescence overlapped with SIKE fluorescence?). Significant co‐occurrence is designated by a value of ≥ 0.5 15. Codistribution measured the overlap of fluorescence between SIKE and the cellular marker to assess whether the majority of each protein's fluorescence overlapped, indicated by a Pearson's correlation coefficient (PCC) of ≥ 0.5 15. To identify cytosolic and nuclear puncta, SIKE colocalization with endosomal markers, RNA granules, and nucleolus‐associated proteins was assessed (Figs 4 and 5). No significant co‐occurrence (colocalization coefficient of ≥ 0.5 ± 2 SD, 95% confidence interval) between SIKE and these markers was identified (Fig. 6). The thresholded PCC also did not indicate significant codistribution (PCC value ≥ 0.5, data not shown) with SIKE. From these data, SIKE puncta do not colocalize with classic endosomal markers, RNA granules, or the nucleolus.

**Figure 7:**
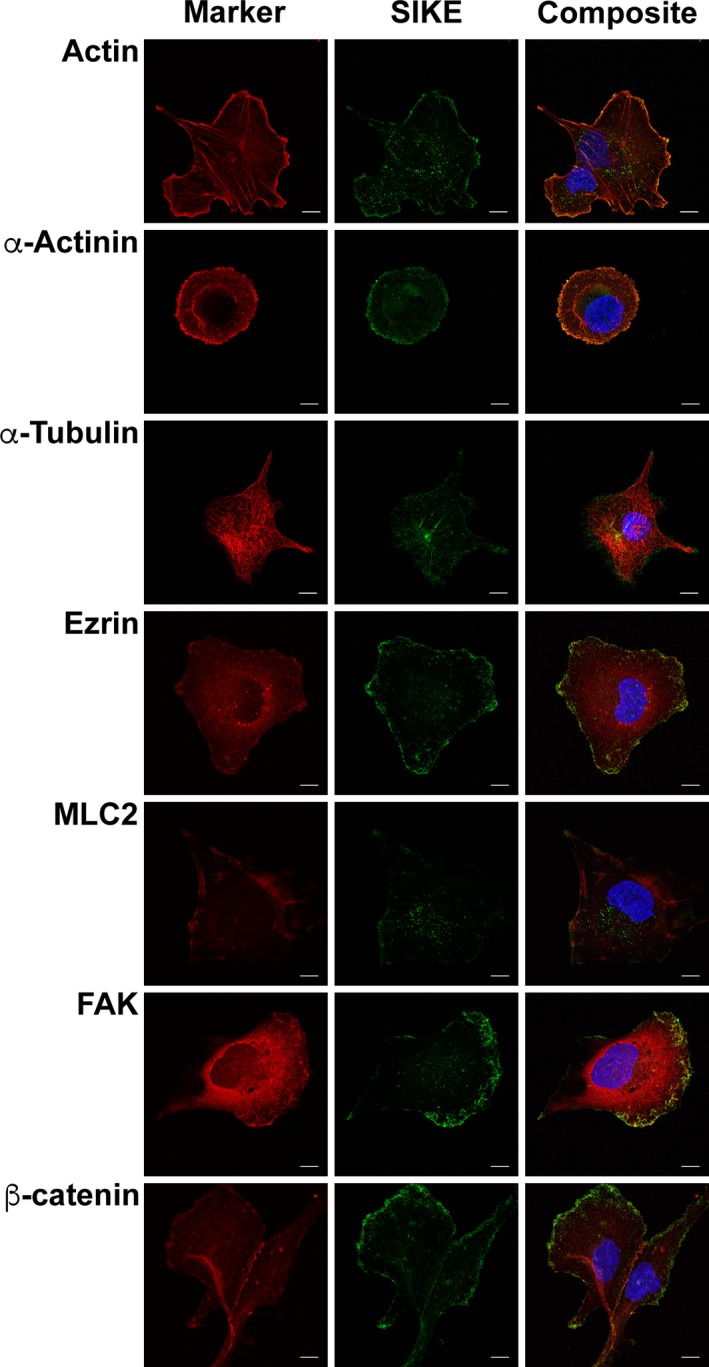
In epithelial cells, SIKE colocalizes with actin cytoskeletal markers. DOV13 cells were fixed, permeabilized, and stained with mouse anti‐cytoskeletal marker antibody as indicated followed by anti‐mouse Alexa Fluor 555 (red), rabbit anti‐SIKE antibody followed by anti‐rabbit Alexa Fluor 488 (green) and Hoechst 33342 (blue). F‐actin was stained with Alexa Fluor 555 Phalloidin. Cytoskeletal markers: actin, α‐actinin, α‐tubulin, ezrin, myosin light chain 2 (MLC2), focal adhesion kinase (FAK), β‐catenin. Images are maximum intensity of *z*‐stack projections. Scale bar, 10 μm. Images are representative of two independent experiments, five images/experiment. Images are maximum intensity *z*‐stack projections. Intersection of fluorescence for SIKE to target was significant (≥ 0.5) for actin, α‐actinin, and ezrin.

**Figure 8:**
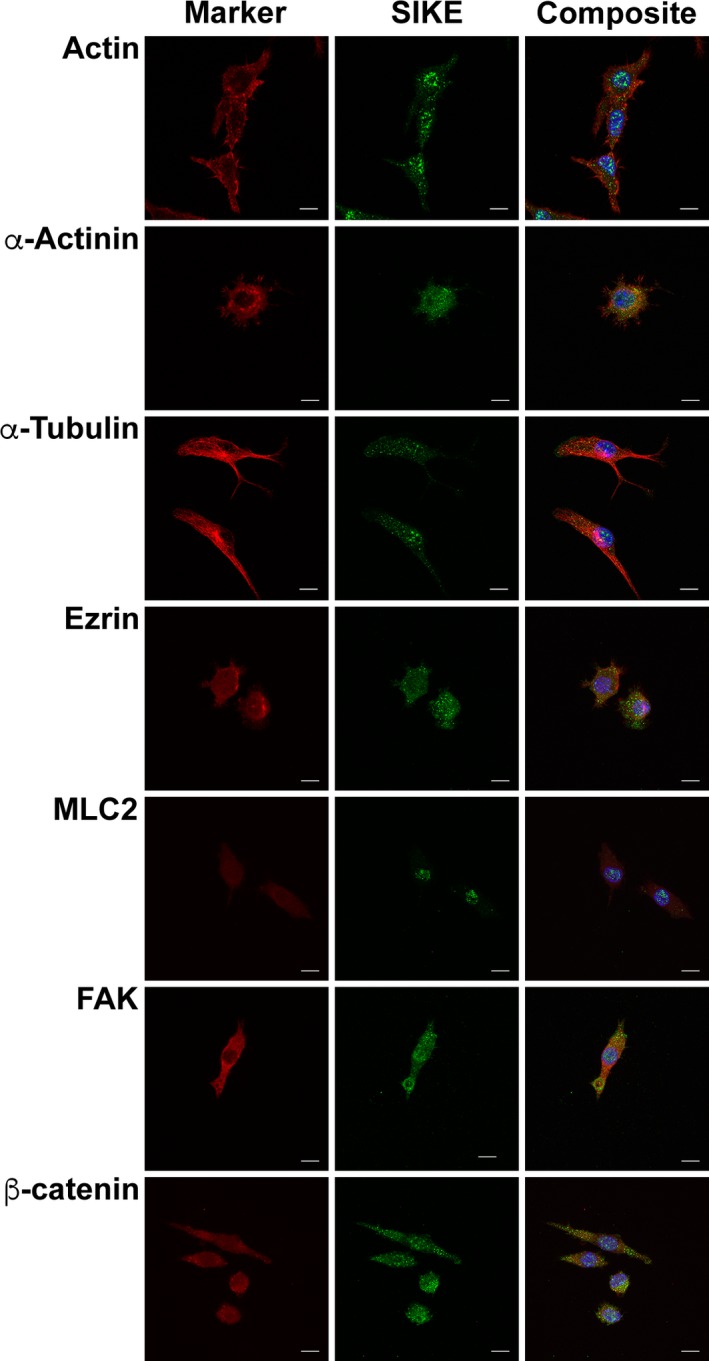
In myeloid cells, SIKE colocalizes with actin and microtubule markers. RAW264.7 cells were fixed, permeabilized, and stained with mouse anti‐cytoskeletal marker antibody as indicated followed by anti‐mouse Alexa Fluor 555 (red), rabbit anti‐SIKE antibody followed by anti‐rabbit Alexa Fluor 488 (green) and Hoechst 33342 (blue). F‐actin was stained with Alexa Fluor 555 Phalloidin. Cytoskeletal markers: actin, α‐actinin, α‐tubulin, ezrin, MLC2, focal adhesion kinase (FAK), β‐catenin. Images are maximum intensity of *z*‐stack projections. Scale bar, 10 μm. Images are representative of two independent experiments, five images/experiment. Images are maximum intensity *z*‐stack projections. Intersection of fluorescence for SIKE to target was significant (≥ 0.5) for α‐actinin.

**Figure 9:**
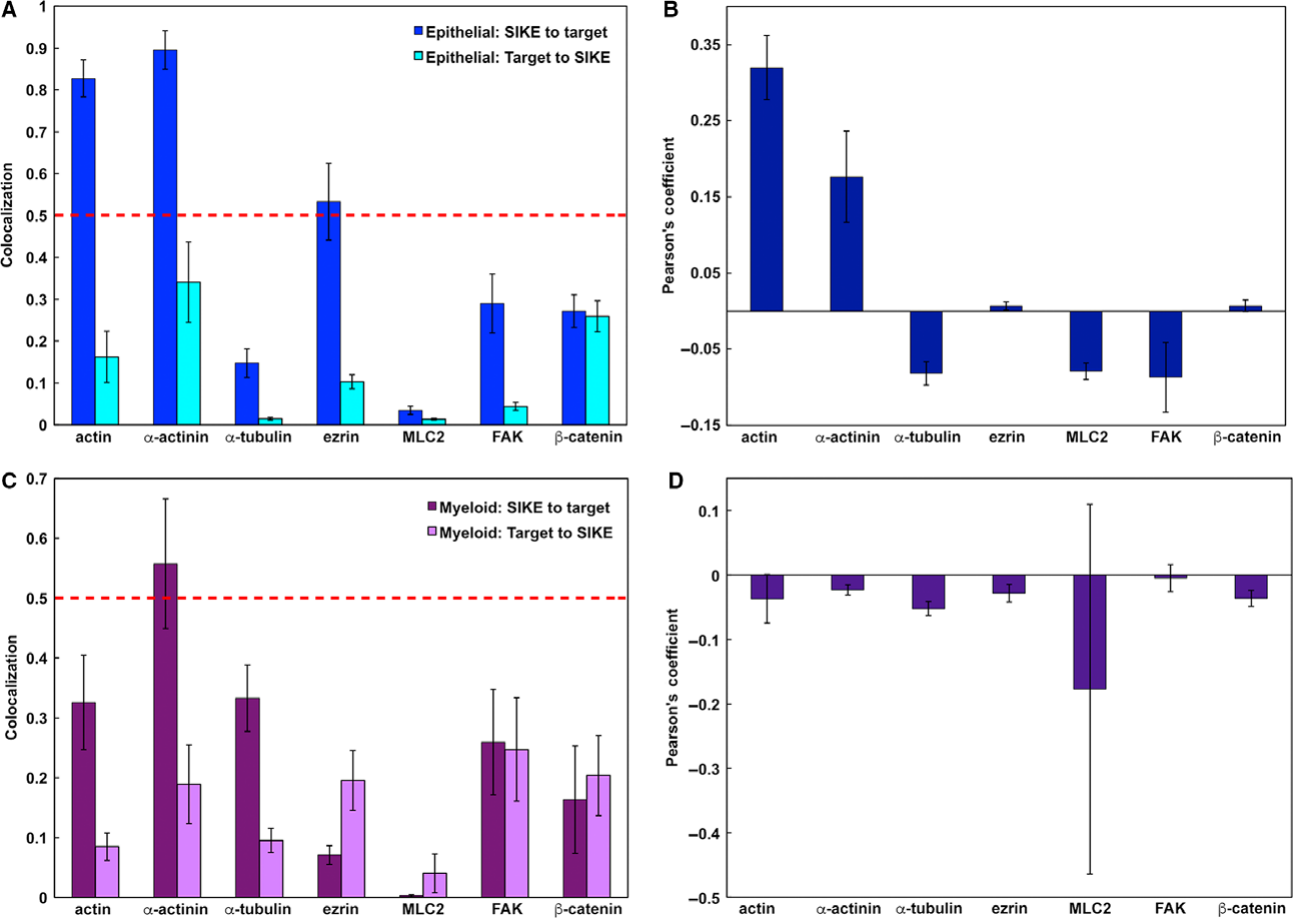
Quantitation of colocalization between SIKE and cytoskeletal markers. volocity software (PerkinElmer) was used to define SIKE or cytoskeletal marker volumes on thresholded images. Fraction of intersecting volumes was determined for SIKE to marker compared to total SIKE volume and marker to SIKE compared to total marker volume (A, C). These graphs represent co‐occurrence. Significant co‐occurrence was noted when values were > 0.5 with a 95% confidence interval. Thresholded PCC was determined (B, D) to assess codistribution of SIKE with cytoskeletal markers. Error bars represent standard error, *n* = 5. (A) In epithelial cells, co‐occurrence of actin, α‐actinin, and ezrin was observed for SIKE fluorescence. In myeloid cells, co‐occurrence of α‐actinin was observed for SIKE fluorescence. No significant (≥ 0.5, as noted in Ref. 27) codistribution of cytoskeletal marker and SIKE fluorescence was observed for either epithelial (B) or myeloid (D) cells.

In addition to reduced migration when SIKE expression is knocked out, endogenous SIKE immunofluorescence and co‐immunoprecipitation tandem MS/MS data indicated SIKE associated with cytoskeletal structures and proteins, respectively. To determine which components of the cytoskeleton interact with SIKE, colocalization of SIKE with several cytoskeletal markers was examined (Figs 7 and 8). In epithelial cells (Fig. 7), co‐occurrence of SIKE fluorescence (colocalization ≥ 0.5, blue bars) with actin, α‐actinin, and ezrin fluorescence was observed, whereas no cytoskeletal marker fluorescence showed significant co‐occurrence (colocalization ≥ 0.5, cyan bars) with SIKE fluorescence (Fig. 9A) as measured by volocity software. Thresholded PCC analysis indicated no significant codistribution of SIKE and cytoskeletal markers (Fig. 9B). To share significant codistribution between two markers, the PCC should be in the range of ≥ 0.5–1. In myeloid cells (Figs 8 and 9C), significant intersection of SIKE fluorescence (colocalization ≥ 0.5, purple bars) with α‐actinin was retained, but was reduced compared to epithelial cells. Although below the value for significant colocalization (≥ 0.5), intersection of SIKE fluorescence with α‐tubulin was increased twofold, while SIKE to ezrin intersection was decreased fivefold in myeloid cells. The thresholded PCC analysis indicated a near zero value for all markers corresponding to no significant codistribution between SIKE and marker fluorescence. From this analysis, the greatest percentage of SIKE fluorescence intersected with α‐actinin in both epithelial (90 ± 5%) and myeloid (56 ± 11%) cells types.

### SIKE interacts with α‐actinin and tubulin, but not actin in reciprocal immunoprecipitations

**Figure 10:**
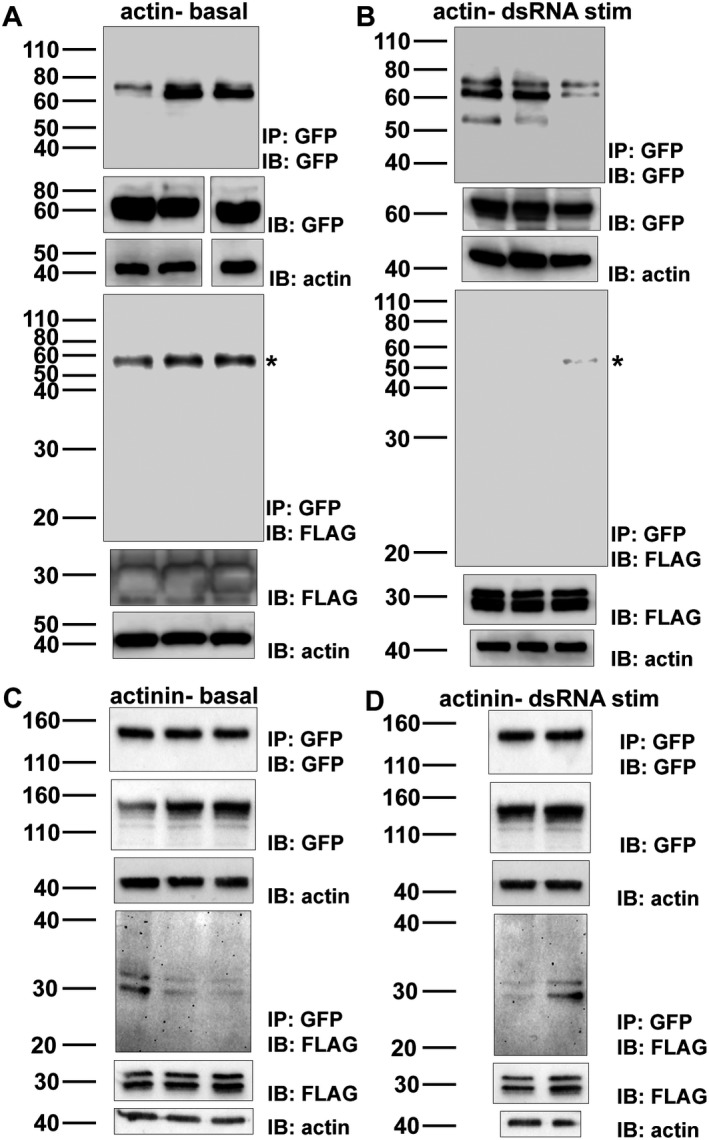
α‐Actinin, but not actin, immunoprecipitates SIKE. Five hundred microgram of protein from six independent transfections of HEK293 cells with pmEGFP‐β‐actin (A, B) or pEGFP‐α‐actinin (C, D) and pCMV5a‐SIKE‐FLAG was exposed to protein G Dynabeads charged with anti‐GFP antibody that had been cross‐linked to beads with bis(sulfosuccinimidyl)suberate as per manufacturers’ protocol. Proteins were eluted from beads into 1× NuPAGE SDS/PAGE sample buffer and were separated by Tris/glycine SDS/PAGE, transferred to nitrocellulose, and immunoblotted for the cytoskeleton protein and FLAG. Three lysates were from cells that had been stimulated with 50 μg·mL ^−1^ polyriboinosinic : polyribocytidylic acid (dsRNA) 12 h prior to harvesting to mimic a viral infection and induce SIKE phosphorylation. Actin served as a loading control. In each panel, (top) IP/IB of cytoskeletal protein with IB of lysate for cytoskeletal protein control and loading control; (bottom) IP of cytoskeletal protein IB of SIKE with IB of lysate for SIKE protein control and loading control. (A) basal state immunoprecipitation of actin, (B) dsRNA‐stimulated immunoprecipitation of actin, (C) basal state immunoprecipitation of α‐actinin, (D) dsRNA‐stimulated immunoprecipitation of α‐actinin. Molecular weights of proteins: SIKE‐FLAG (27 kDa), EGFP‐actin (67 kDa), and EGFP‐α‐actinin (132 kDa). *Nonspecific band observed with anti‐FLAG antibody. Blots are representative of at least 5–6 independent experiments (2–3 shown).

**Figure 11:**
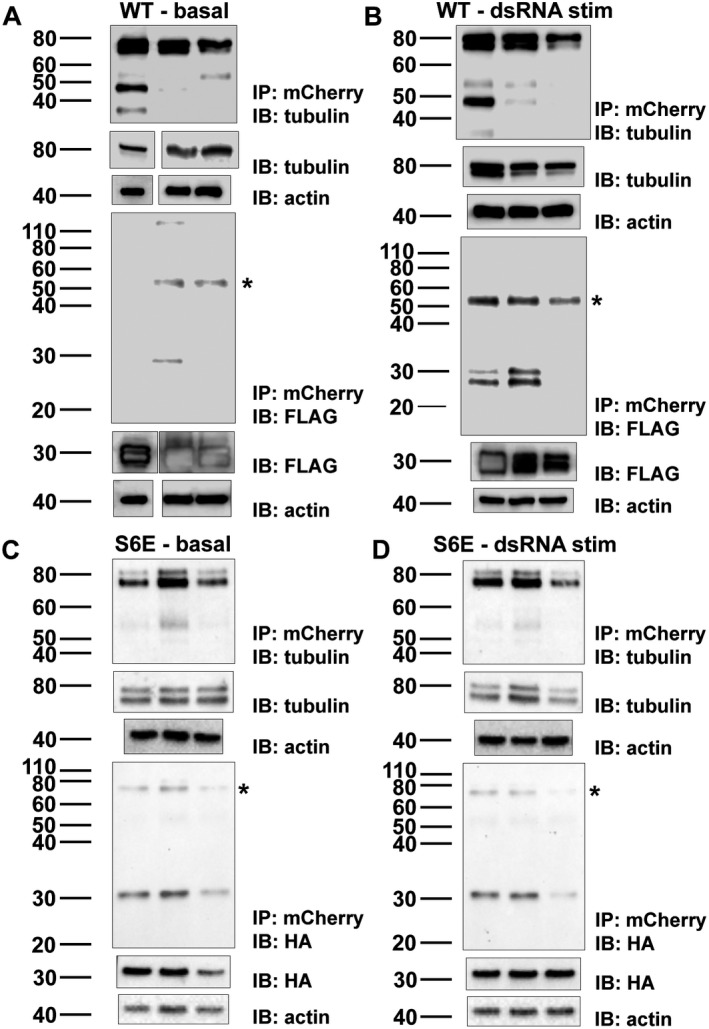
SIKE phosphorylation enhances interaction with tubulin. Five hundred microgram of protein from six independent transfections of HEK293 cells with pmCherry‐α‐tubulin and pCMV5a‐SIKE‐FLAG (A, B) or phosphomimetic SIKE pCMVHA‐SIKE S6E (S133/185/187/188/190/198E) (C, D) was exposed to protein G Dynabeads charged with anti‐mCherry antibody that had been cross‐linked to beads with bis(sulfosuccinimidyl)suberate as per the manufacturers’ protocol. Proteins were eluted from beads into 1× NuPAGE SDS/PAGE sample buffer and were separated by Tris/glycine SDS/PAGE, transferred to nitrocellulose, and immunoblotted for tubulin and FLAG or HA (SIKE). Three lysates were from cells that had been stimulated with 50 μg·mL^−1^ polyriboinosinic : polyribocytidylic acid (dsRNA) 12 h prior to harvesting to mimic a viral infection and induce SIKE phosphorylation. Actin served as a loading control. In each panel, (top) IP/IB of tubulin with IB of lysate for tubulin protein control and loading control; (bottom) IP of tubulin IB of SIKE with IB of lysate SIKE protein control and loading control. (A) basal state immunoprecipitation of tubulin with WT SIKE, (B) dsRNA‐stimulated immunoprecipitation of tubulin with WT SIKE, (C) basal state immunoprecipitation of tubulin with S6E SIKE, (D) dsRNA‐stimulated immunoprecipitation of tubulin with S6E SIKE. Molecular weights of proteins: FLAG or HA SIKE (27 kDa), and mCherry tubulin (80 kDa). *Nonspecific band observed with (A, B) anti‐FLAG antibody at ~ 55 kDa and (C, D) anti‐HA antibody at ~ 70 kDa. Blots are representative of at least six independent experiments (three shown).

As MS and colocalization data suggested interactions with cytoskeletal elements, actin‐based filaments and/or tubulin/microtubules, reciprocal immunoprecipitation reactions using a cytoskeletal protein, actin or α‐actinin for actin‐based filaments or tubulin for microtubules, as bait were completed. Actin, α‐actinin, or tubulin, tagged with either eGFP or mCherry, was transiently transfected into HEK293 cells with either FLAG‐SIKE or phosphomimetic (S6E) HA SIKE. Using magnetic bead technology, the cytoskeletal proteins were immunoprecipitated from the lysates with either an anti‐GFP or anti‐mCherry antibody cross‐linked to the protein G‐labeled beads and precipitated networks assessed for cytoskeletal protein and SIKE. SIKE only lysates, used as controls to test for nonspecific SIKE interactions to either protein G‐labeled beads or antibody‐labeled beads, showed no interaction (data not shown). SIKE and GFP lysates (no labeled cytoskeletal proteins), used as a control to test for nonspecific SIKE interactions to GFP, showed no interaction (data not shown). SIKE : cytoskeletal protein interactions were tested under basal and dsRNA, which induces phosphorylation of SIKE 9, conditions. For each condition, replicates of the 5–6 independent experiments are shown. Under basal or dsRNA‐stimulated conditions, SIKE was not immunoprecipitated with actin (Fig. 10A,B). In contrast, SIKE : α‐actinin interactions were observed ± dsRNA stimulation (Fig. 10C,D). Using WT FLAG‐SIKE, the SIKE : tubulin interaction was enhanced following dsRNA stimulation with two of three reactions revealing a SIKE : tubulin complex (Fig. 11B), whereas under basal conditions only one of three reactions indicated a SIKE : tubulin interaction (Fig. 11A). These data suggested that dsRNA stimulation altered either the components of the complex or the environment to enhance the SIKE : tubulin complex. We had previously shown that dsRNA stimulation enhances SIKE phosphorylation, but the six phosphorylation sites of SIKE are not equally modified, even in *in vitro* reactions 9. To determine if the SIKE : tubulin interaction was dependent upon SIKE phosphorylation, we used a SIKE phosphomimetic (S6E) construct to complete the reciprocal immunoprecipitation reactions with tubulin (Fig. 11C,D). Under both basal and dsRNA‐stimulated conditions, all reactions showed a phosphomimetic SIKE : tubulin complex consistent with SIKE phosphorylation enhancing the SIKE : tubulin interaction.

### SIKE interacts directly with tubulin and α‐actinin

**Figure 12:**
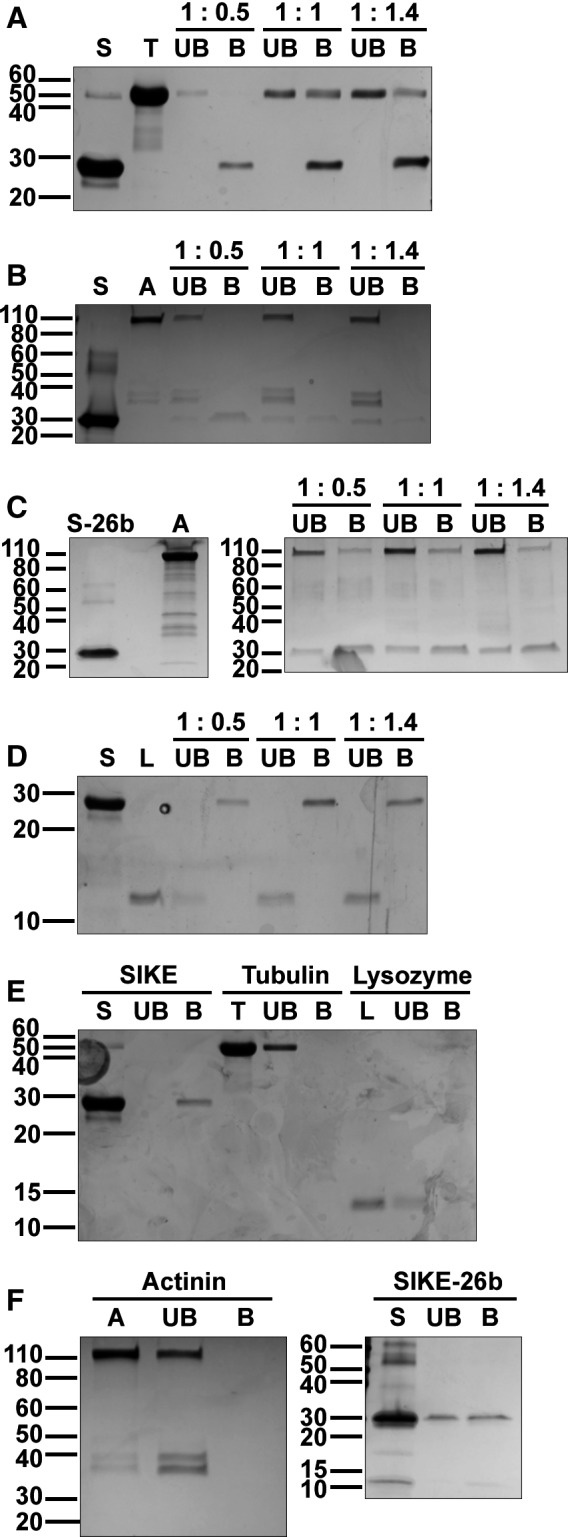
SIKE forms direct interactions with tubulin and α‐actinin. IVP reactions of 6xHis‐SIKE‐FL (SIKE or S) or FL‐SIKE‐6xHis (SIKE‐26b or S‐26b) (8 μm) with monomeric tubulin (A, 4–11.2 μm), α‐actinin (B, C, 4–11.2 μm), or lysozyme (D, 4–11.2 μm) were precipitated with Ni‐NTA resin and separated by SDS/PAGE (12% (A), 4–15% (B, C), or 15% (D) Tris/glycine) and stained with SimplyBlue SafeStain. Control reactions of individual proteins incubated with Ni‐NTA resin were subjected to the same reaction conditions and separated by SDS/PAGE (15% (E), 4–15% (F)). UB, unbound sample; B, bound sample; 1 : 0.5 molar ratio, 1 : 1 molar ratio, 1 : 1.4 molar ratio. (A) Lanes: 1 – SIKE‐FL starting material; 2 – tubulin starting material; 3 – 1 : 0.5 UB; 4 – 1 : 0.5 B; 5 – 1 : 1 UB; 6 – 1 : 1 B; 7 – 1 : 1.4 UB; 8 – 1 : 1.4 B. B) Lanes: 1 – SIKE‐FL starting material; 2 – α‐actinin starting material; 3 – 1 : 0.5 UB; 4 – 1 : 0.5 B; 5 – 1 : 1 UB; 6 – 1 : 1 B; 7 – 1 : 1.4 UB; 8 – 1 : 1.4 B. (C) Gel 1 Lanes: 1 – SIKE‐26b starting material; 2 – blank; 3 – α‐actinin starting material. Gel 2 Lanes: 1 – 1 : 0.5 UB; 2 – 1 : 0.5 B; 3 – 1 : 1 UB; 4 – 1 : 1 B; 5 – 1 : 1.4 UB; 6 – 1 : 1.4 B. D) Lanes: 1 – SIKE‐FL starting material; 2 – lysozyme starting material; 3 – 1 : 0.5 UB; 4 – 1 : 0.5 B; 5 – 1 : 1 UB; 6 – 1 : 1 B; 7 – 1 : 1.4 UB; 8 – 1 : 1.4 B. (E) Lanes: 1 – SIKE‐FL starting material; 2 – UB; 3 – B; 4 – tubulin starting material; 5 – UB; 6 – B; 7 – lysozyme starting material; 8 – UB; 9 – B. F) Gel 1 Lanes: 1 – α‐actinin starting material; 5 – UB; 6 – B. Gel 2 Lanes: 1 – SIKE‐26 starting material; 2 – UB; 3 – B; control reactions show that only SIKE is bound to the Ni‐NTA resin. The C‐terminal 6xHis‐tagged SIKE is not all bound to the Ni‐NTA, but can be found in the unbound and bound fractions. SIKE does not interact nonspecifically with the negative control, lysozyme. In tubulin reactions, SIKE precipitates tubulin indicating a direct interaction. When SIKE is 6xHis‐tagged at the N terminus, α‐actinin pre‐incubation inhibits SIKE's ability to bind to the resin, suggesting α‐actinin may complex to SIKE and mask SIKE's 6xHis tag. With a C‐terminal 6xHis tag, SIKE precipitates α‐actinin indicating a direct interaction. Gels are representative of three independent experiments.

As the reciprocal immunoprecipitations could not distinguish between direct or indirect interactions between SIKE and the cytoskeletal proteins, IVP reactions were completed. Recombinant 6xHis‐tagged SIKE and cytoskeletal protein or lysozyme (negative control) were combined in 1 : 0.5, 1 : 1, or 1 : 1.4 molar ratios. SIKE and associated proteins were precipitated from the reaction with Ni‐NTA agarose beads (bound (B) sample). Supernatant removed from the pelleted Ni‐NTA beads represented the unbound (UB) fraction. Starting material, bound, and unbound samples were assessed via SDS/PAGE (Fig. 12). Control reactions (Fig. 12E,F) showed that SIKE bound to the beads, but neither tubulin, α‐actinin, nor lysozyme bound nonspecifically to the beads. The negative control, lysozyme, did not form a complex with SIKE (Fig. 12D). In SIKE : tubulin reactions (Fig. 12A), SIKE : tubulin interactions were observed in the bound (B) samples of the 1 : 1 and 1 : 1.4 reactions. The SIKE : α‐actinin reactions using N‐terminal 6xHis‐tagged SIKE showed that α‐actinin remained in the unbound fraction (Fig. 12B). More importantly, as the α‐actinin concentration increased, the intensity of the SIKE band in the bound fraction decreased and a SIKE band appeared in the unbound fraction and increased in intensity with increasing α‐actinin concentration. This suggested that the SIKE : α‐actinin interaction interfered with the N‐terminal 6xHis tag of SIKE binding to the Ni‐NTA resin. When a C‐terminal 6xHis‐tagged SIKE was used, α‐actinin was precipitated with SIKE (Fig. 12C).

## Discussion

Suppressor of IKKepsilon function within the innate immune response remains an open question. In studies using overexpression of SIKE, TBK1‐mediated activation of either IRFs or Akt is inhibited 8, 10, leading to the hypothesis that SIKE functioned as an endogenous inhibitor of TBK1 activity. Yet our previous studies demonstrated that SIKE is a TBK1 substrate such that the observed ‘inhibition’ is a consequence of SIKE's ability to alter the downstream effect of TBK1 activity through its high‐affinity interaction as a substrate with the kinase 9. Identification of SIKE within the supramolecular STRIPAK complex 11 that, in flies, regulates a kinase cassette responsible for final tissue and organ size 13, illustrates more diverse functions for SIKE than altering TBK1 activity. In this study, we used a combination of a SIKE knockout cell line and investigation of the SIKE interaction network to define SIKE function through association and demonstrated for the first time that SIKE directly interacts with cytoskeletal proteins.

To garner insight into SIKE's biological function, a SIKE null cell line was created and assessed. Loss of SIKE protein expression, confirmed by immunoblot, gave no gross phenotype in terms of cell morphology. As SIKE has been linked to the antiviral innate immune response 8 and several host defenses in response to innate immune stimuli require cytoskeletal rearrangement 16, we employed a simple scratch assay to examine a classic example of cytoskeletal rearrangement, cell migration. Compared to the parental cell line, SIKE null cells showed significantly reduced migration. These data suggested that SIKE may function in cytoskeletal rearrangements that support processes involved in cell migration.

As processes involved in cell migration such as protrusion and adhesion, translocation of the cell body, and retraction entail large macromolecular complexes, the SIKE interaction network was surveyed through co‐immunoprecipitation of FLAG‐tagged SIKE followed by tandem MS/MS to assess if SIKE was associated with a distinct complex (e.g., focal adhesions). The study identified cytoskeletal proteins, chaperones, nucleic acid binding proteins, and enzymes as SIKE interaction partners. As most of the identified SIKE interaction partners were consistent with a contaminant database based upon our epitope tag and methodology, CRAPome expt. CC66 17, we developed a workflow, including immunofluorescence assays and reciprocal immunoprecipitations, to validate SIKE interaction partners.

We examined endogenous SIKE localization in two cell types that would be most relevant to innate immunity, myeloid (progenitor of several immune cell types) and epithelial (lining of surfaces exposed to environment and potential site of first contact with pathogen). Endogenous localization of SIKE between myeloid and epithelial cells differed. Epithelial cells revealed distinctive staining patterns consistent with stress fibers and lamellipodia that would support interactions with cytoskeletal proteins. Additionally, SIKE was found in cytosolic puncta. In contrast, myeloid cells lacked pronounced staining of cytoskeletal structures, but revealed both cytosolic and nuclear puncta. Nuclear staining would be compatible with the subset of nucleic acid binding partners identified by MS/MS, but is also consistent with a previous report showing SIKE interacted with emerin, a nuclear envelope‐associated protein that stabilizes and promotes the formation of a nuclear actin cortical network 18. To further refine the structures with which endogenous SIKE associated, colocalization using a panel of endosomal, nucleic acid, and cytoskeletal markers was assessed.

Neither the endosomal nor the nucleic acid markers yielded significant colocalization with SIKE in either cell type, leaving the identity of the cytosolic and nuclear puncta unknown, but excluding a number of classic cellular structures. Cytoskeletal colocalization markers, on the other hand, resulted in significant intersecting fluorescence with SIKE. We first examined co‐occurrence, the spatial overlap of SIKE and the cytoskeletal marker from the perspective of both SIKE and the cytoskeletal marker. In epithelial cells, SIKE fluorescence overlapped significantly (colocalization ≥ 0.5) with actin, α‐actinin, and ezrin, whereas the corresponding fluorescence for the cytoskeletal marker did not significantly overlap with SIKE for any cytoskeletal marker. These data suggest that SIKE associated with a subpopulation of these cytoskeletal proteins’ fluorescence. This interpretation was confirmed by thresholded Pearson's coefficient analysis, which measures if two markers not only overlap with one another, but codistribute in proportion to one another within and between structures. A value of 1, 0, or −1 corresponds to perfect positive correlation, no correlation, or perfect negative correlation, respectively. The thresholded Pearson's coefficient indicated a slight positive correlation (0.18 ± 0.06) for only SIKE : α‐actinin colocalization, but, as expected, did not meet the value of ≥ 0.5 to be significant. In myeloid cells, SIKE fluorescence only significantly overlapped with α‐actinin, but evaluation by PCC yielded no positive correlation with any marker. These data support that SIKE distribution significantly colocalizes with actin, α‐actinin, and ezrin in epithelial cells and α‐actinin in myeloid cells, but, when examining the entire population of the cytoskeletal marker fluorescence, only a subset of cytoskeletal marker fluorescence is associated with SIKE.

Reciprocal immunoprecipitations in which the cytoskeletal protein served as bait further confirmed SIKE : cytoskeletal protein interactions. Although colocalization data strongly supported SIKE interactions with actin cytoskeletal components, cell migration data and the LC‐MS/MS data also supported a potential SIKE interaction with microtubule components. Therefore, tubulin was included as a bait protein. Unexpectedly, the data showed that SIKE interacts with α‐actinin and tubulin, but not actin. The actin/α‐actinin data are consistent with α‐actinin function, which cross‐links actin filaments 19. In the LC‐MS/MS and colocalization data, the results suggest that α‐actinin bridged the perceived SIKE : actin interaction by α‐actinin's close association with actin. In the absence of enough α‐actinin, such as transient expression of the eGFP‐labeled actin for reciprocal immunoprecipitation, to bridge a SIKE : actin interaction, no SIKE : actin interaction was observed. Of note, *in vivo*, phosphomimetic SIKE enhanced the tubulin : SIKE interaction independent of dsRNA stimulation, suggesting that the inconsistent SIKE : tubulin interaction observed under basal and dsRNA‐stimulated conditions with WT SIKE may have been due to different levels of SIKE phosphorylation under these conditions. When examined by IVP, the direct, ‘weaker’ WT SIKE : tubulin interaction was confirmed. This result does not exclude that SIKE phosphorylation may enhance this interaction. The IVP reaction for α‐actinin suggested α‐actinin may interact near the N terminus of SIKE. With the N‐terminally 6xHis‐tagged SIKE, as the molar ratio of α‐actinin in the SIKE : α‐actinin reaction was increased, the amount of 6xHis‐tagged SIKE bound to the Ni‐NTA was reduced, in contrast to the SIKE alone reaction, which readily bound to the Ni‐NTA resin. In the experimental setup, the SIKE is pre‐incubated with α‐actinin prior to precipitation by Ni‐NTA. These results suggested that an α‐actinin interaction with SIKE blocked SIKE's 6xHis tag from interacting sufficiently with the Ni‐NTA resin thereby reducing precipitation of SIKE. To explore this possibility, we repeated the IVP with a C‐terminal 6xHis‐tagged SIKE. This construct was capable of precipitating α‐actinin. This direct interaction with α‐actinin is consistent with the immunofluorescence assays. These data implicate SIKE in both actin‐based cytoskeletal structure associated with α‐actinin as well as microtubules, dependent upon the phosphorylation state of SIKE.

The link between SIKE and the cytoskeleton is of particular interest as it contributes to a hypothesis for SIKE function: SIKE acts as a bridge between cytoskeletal structures and innate immune signaling pathways. The well established SIKE : TBK1 interaction 8, 9, 10 places SIKE in contact with a catalytic hub, TBK1, which signals in innate immune responses, bacterial sequestration and elimination, and several pathological conditions such as obesity, glaucoma, and cancer 20, 21, 22. The reduced migration of SIKE null cells suggests that SIKE functions in processes involving cytoskeletal rearrangement, and the phosphorylation enhanced interaction between SIKE and tubulin may signify a switch in SIKE interactions sensitive to pathogen sensing via TBK1‐mediated phosphorylation. TBK1 activation and substrate specificity have both been linked to localization (reviewed in Ref. 22) that, in turn, emphasizes the significance of binding partners to direct TBK1 localization. This relationship has already been established for TBK1 binding partners, optineurin and NAK1‐associated protein 1 (NAP1), that direct TBK1 to autophagosomes 6 and cytosolic puncta 23, respectively. Further, SIKE's ability to function as a bridge has been established in the Drosophila STRIPAK complex where the SIKE homolog connects striatin within the fly STRIPAK complex to the complex's target, Hpo kinase. This work raises several questions regarding the function of SIKE including how association with α‐actinin or tubulin alter cytoskeletal rearrangements to enhance or regulate these processes and whether this association with cytoskeletal proteins enhances the function of the target cytoskeletal protein or delivers SIKE‐associated proteins to these cytoskeletal structures. Rather than acting as an endogenous inhibitor of TBK1, at physiological concentrations, SIKE may function to localize TBK1's catalytic activity where it is required.

Overall, we have defined a SIKE interaction network that directly links SIKE to the cytoskeletal proteins, tubulin and α‐actinin. The SIKE : tubulin interaction is sensitive to the phosphorylation state of SIKE providing a means to regulate this interaction and sensitize this interaction to environmental cues. In conclusion, our results show that SIKE is capable of making multiple protein : protein interactions that link key catalytic activity to the cytoskeletal structure.

## Materials and methods

### Materials

All chemicals purchased from Sigma‐Aldrich (St. Louis, MO, USA) unless otherwise stated. Cell culture reagents were purchased from Thermo Fisher Scientific (Waltham, MA, USA) unless otherwise noted.

### Cell culture

DOV13 (X. Fang, Virginia Commonwealth University, Richmond, VA, USA), HEK293 (ATCC, Manassas, VA, USA) and RAW264.7 (ATCC) cells were cultured in complete media (RPMI 1640 supplemented with 10% low‐endotoxin FBS, 20 mm l‐glutamine, 100 mm HEPES, 10 mm sodium pyruvate, 1× nonessential amino acid solution, 100 μg·mL^−1^ penicillin, and 100 U·mL^−1^ streptomycin) at 37 °C in a 5% (v/v) CO_2_ humidified environment. HAP1 and HAP1 CRISPR/Cas9 SIKE knockout (SIKE‐CR) cell lines (Horizon Discovery, Cambridge, UK) were cultured in complete Iscove's modified Dulbecco's medium (IMDM) supplemented with 10% low‐endotoxin FBS, 100 μg·mL^−1^ penicillin, and 100 U·mL^−1^ streptomycin) at 37 °C in a 5% (v/v) CO_2_ humidified environment as per manufacturer's protocol. The HAP1 cell line is a near‐haploid human cell line of myeloid origin (immune cell lineages) that was derived from the male chronic myelogenous leukemia cell line KBM‐7 24. The SIKE‐CR cell line was created by Horizon Discovery from the HAP1 parental cell line using a CRISPR/Cas9 approach to introduce a frame shift mutation by deleting 5 nte from bp 173‐177 in exon 2 of the coding sequence (NM_025073). This frameshift was sequence‐confirmed and resulted in a premature stop codon following residue 78.

### Scratch assays and analyses

HAP1 or SIKE‐CR cells were seeded in 35‐mm dishes at 2.5 × 10^6 ^cells·mL^−1^ in incomplete IMDM (IMDM supplemented with 0.5% FBS, 100 μg·mL^−1^ penicillin, and 100 U·mL^−1^ streptomycin) and allowed to adhere overnight at 37 °C in a 5% CO_2_ (v/v) humidified environment. Cell monolayer was assessed for confluency, and a scratch was introduced to the monolayer with a sterile 200‐μL pipette tip held perpendicular to plate surface. Medium was removed, and cells were washed 3× with 1× PBS and then replenished with complete IMDM. Bright‐field mages of the scratched area were taken every 10 min for 24 h with a 10× objective on an EVOS FL Cell Imaging System (Thermo Fisher Scientific). An ibidi Gas Incubation System (Martinsried, Germany) was used to maintain incubation conditions at 37 °C, 5% CO_2_, and 90% humidity. A minimum of three replicates per cell line was completed.

Analysis of scratch closure was performed using the application ‘SketchAndCalc Area Calculator’ (http://www.SketchAndCalc.com). The entire image area and area of scratch were determined. At time zero, the percentage of area filled was calculated. At each subsequent time point, the percentage of area filled was calculated and subtracted from the time zero value to determine the percentage of scratch closure. Two analysis strategies were used to assess cell migration. The first method was a full image analysis; this strategy provided an analysis of the entire scratched area. The second method was a 300‐pixel analysis; the image was cropped 300 pixels from the cell/scratch boundary and scratch closure assessed on this defined area. As not all scratches were of uniform width, the 300 pixel analysis gave a standardized migration distance. Student's *t*‐test (unpaired, 95% confidence interval, two‐tailed) was used to assess HAP1 and SIKE‐CR data for significant differences in cell migration.

### Transfections

#### Mass spectrometry samples

Cells were seeded in triplicate on a six‐well plate at 0.4 × 10^6 ^cells·mL^−1^ and transfected with 4 μg per well WT SIKE (pCMVFLAG5a SIKE 6xHis) using Lipofectamine 2000 (Thermo Fisher Scientific) as per manufacturer's protocol. For dsRNA‐stimulated samples, 21 h after transfection, cells were stimulated with 50 μg·mL^−1^ polyriboinosinic : polyribocytidylic acid (pI : pC) in complete medium for 3 h. Cells were harvested 24 h after transfection by washing from plate and pelleted by centrifugation (3000 ***g***, 3 min, 4 °C).

#### Cytoskeletal protein immunoprecipitation samples

A total of 1 × 10^6^ cells were seeded in triplicate on 60‐cm^2^ plates and transfected with 24 μg of total DNA consisting of either a 1 : 1 ratio of pCMVFLAG5a SIKE 6xHis or phosphomimetic SIKE (pCMVHA‐SIKE S6E (S133, 185, 187, 188, 190, 198E) 9) and DNA of cytoskeletal protein (pmEGFP‐β‐actin, pmCherry‐α‐tubulin, or pEGFP‐α‐actinin (Addgene, Cambridge, MA, USA)) or individual constructs plus an empty vector (pCDNA 3.1) using Lipofectamine 2000 according to manufacturer's protocol (Thermo Fisher Scientific). After 48 h, transfection efficiency was assessed by fluorescence microscopy. Only plates with > 50% transfection efficiency were used for co‐immunoprecipitation studies. Where indicated, cells were stimulated with 50 μg·mL^−1^ pI : pC at the 36 h post‐transfection. Cells were harvested at 48 h by washing from plate and pelleted by centrifugation (3000 ***g***, 3 min, 4 °C).

#### For MS and co‐IP samples

Cells were resuspended in lysis buffer (0.02 m HEPES, pH 7.4, 0.15 m NaCl, 10 mm NaF, 2 mm DTT, 2 mm EGTA, 1.5 mm MgCl_2_, 1 mm Na_3_VO_4_, 2.7 mg·mL^−1^ β‐glycerophosphate, 1 mg·mL^−1^ *n*‐ethylmaleimide, 0.5% Triton X‐100, and 1× complete EDTA‐free protease inhibitor cocktail) and sonicated to ensure complete cell lysis. Lysates were cleared by centrifugation (14 000 ***g***, 30 min, 4 °C). Protein concentration of lysate was quantified by the Bradford method (Bio‐Rad, Hercules, CA, USA).

### Immunoprecipitations

#### Mass spectrometry samples

Immunoprecipitated lysate (5 mg protein) with 50 μL of anti‐FLAG M2 affinity gel following manufacturer's protocol. Briefly, lysate was incubated with resin overnight at 4 °C under rotation, washed with 1× TBS (three times) and eluted from resin with 100 μg·mL^−1^ FLAG peptide.

#### Cytoskeletal protein immunoprecipitation samples

7.5 μg of mouse anti‐GFP (BioLegend, San Diego, CA, USA) or rabbit anti‐mCherry (Thermo Fisher Scientific) antibody were cross‐linked to 50 μL of magnetic Dynabeads protein G (Thermo Fisher Scientific) with BS3 cross‐linking reagent (Thermo Fisher Scientific) as per manufacturer's instruction. The incubation time was extended to 1 h at 4 °C, and the quenching was extended to 30 min. Lysates (500 μg of protein) were applied to the bead–antibody complex and allowed to incubate with rotation at 4 °C overnight. Beads were washed in triplicate with eight beads volumes of 1× TBS containing 0.1% Tween‐20 (TBS/Tween). The beads were resuspended in 100 μL of TBS/Tween and transferred to a fresh microcentrifuge tube to avoid eluting proteins bound to the tube wall. Immunoprecipitated proteins were released from the antibody bead complex by heating in 30 μL 1× SDS loading buffer and supernatant transferred to a fresh microcentrifuge tube.

### Mass spectrometry

#### Sample prep

Mass spectrometry samples were diluted to 150–200 μL with 100 mm ammonium bicarbonate to reduce the concentration of salts prior to digestion. The samples were reduced with 5 μL of 10 mm dithiothreitol in 0.1 m ammonium bicarbonate at room temperature for 0.5 h. Then samples were alkylated with 5 μL 50 mm iodoacetamide in 0.1 m ammonium bicarbonate at room temperature for 0.5 h. The samples were digested with 1 μg trypsin overnight and then quenched with 5% (v:v) glacial acetic acid. Fifty microlitre of each sample was concentrated down to 20 μL, and 3–7 μL of the final solution was injected for analysis.

#### LC‐MS

The LC‐MS system consisted of a Thermo Electron LTQ Orbitrap hybrid mass spectrometer system with a nanospray ion source interfaced to a Waters SCX trap column and a Waters NanoAcquity C18 reversed‐phase capillary column. Seven microlitre of the final solution was injected onto the trap column, and the peptides were eluted from the column by an acetonitrile/0.1% formic acid gradient at a flow rate of 0.4 μL·min^−1^ over 60 min. The nanospray ion source was operated at 3.5 kV. The digests were analyzed using the double play capability of the instrument acquiring full scan mass spectra to determine peptide molecular weights and product ion spectra to determine amino acid sequence in sequential scans. This mode of analysis produces approximately 10 000 CAD spectra of ions ranging in abundance over several orders of magnitude. Not all CAD spectra are derived from peptides.

#### Database searching

The data were analyzed by database searching using the Sequest search algorithm against the IPI Human database. Charge state deconvolution and deisotoping were not performed. All MS/MS samples were analyzed using Sequest (XCorr Only) (Thermo Fisher Scientific, San Jose, CA, USA; version 1.2.0.206) and X!Tandem (The GPM, thegpm.org; version CYCLONE (2010.12.01.1)). Sequest (XCorr Only) was set up to search IPI_HUMAN_110411.fasta (92 104 entries) or HUMAN_130430.fasta (89 430 entries—WT SIKE run 2) assuming the digestion enzyme trypsin. X! Tandem was set up to search the IPI_HUMAN_110411 database (92 104 entries) or HUMAN 130430.fasta (89 430—WT SIKE run 2) also assuming trypsin digestion. Sequest (XCorr Only) and X!Tandem were searched with a fragment ion mass tolerance of 0.80 Da and a parent ion tolerance of 15 p.p.m. Oxidation of methionine and carbamidomethyl of cysteine were specified in Sequest (XCorr Only) and X!Tandem as variable modifications or Glu→pyro‐Glu of the N terminus, ammonia loss of the N terminus, Gln→pyro‐Glu of the N terminus, oxidation of methionine and carbamidomethyl of cysteine were specified in X!Tandem as variable modifications.

#### Criteria for protein identification

Scaffold (version Scaffold_4.0.1; Proteome Software Inc., Portland, OR, USA) was used to validate MS/MS‐based peptide and protein identifications. Peptide identifications were accepted if they could be established at greater than 95.0% probability by the PeptideProphet algorithm 25. Protein identifications were accepted if they could be established at greater than 90.0–99.9% probability and contained at least 1–2 identified peptides. Protein probabilities were assigned by the Protein Prophet algorithm 26. Proteins that contained similar peptides and could not be differentiated based on MS/MS analysis alone were grouped to satisfy the principles of parsimony. Proteins sharing significant peptide evidence were grouped into clusters.

All mass spectrometry data and analyses were completed by the VCU Chemical and Proteomic Mass Spectrometry core facility. The mass spectrometry proteomics data have been deposited to the ProteomeXchange Consortium via the PRIDE 1 partner repository with the dataset identifier PXD007262.

### Immunofluorescence assays

DOV13 (2.5 × 10^4^ cells·mL^−1^) or RAW264.7 (1 × 10^4^ cells·mL^−1^) were seeded on 22 × 22 mm acid washed cover slips. Twenty‐four hours after seeding, cells were rinsed with 1× PBS, fixed in ice cold 4% paraformaldehyde for 10 min at RT. All staining procedures were performed at room temperature and with gentle rotation unless otherwise indicated. Cells were permeabilized with 0.1% Triton X‐100 in 1× PBS for 10 min. Cells were washed three times with 1× PBS. Cells were blocked in 5% goat sera (Thermo Fisher Scientific) in 1× PBS for 1 h. Two hundred microlitre of primary antibody in 1× PBS and 5% goat sera was placed on parafilm in a humidity chamber; coverslips were placed, inverted, on top of primary antibody solution, and incubated overnight at 4 °C. In dual‐labeled experiments, both primary antibodies (from different species) were added simultaneously. Coverslips were returned to 35‐mm dishes and washed with 1× PBS three times. Coverslips were incubated for 1 h with 1 mL secondary antibody solution. In dual‐labeled experiments, secondary antibodies were added simultaneously. Following incubation with secondary antibodies, cells were washed in 1× PBS three times. For phalloidin staining, 200 μL of diluted AF555 phalloidin was placed on parafilm in a humidity chamber and coverslips were placed, inverted, on top of the diluted phalloidin. Coverslips were incubated for 20 min at room temperature. After incubation, AF555 phalloidin‐stained coverslips were returned to 35‐mm dishes with 1× PBS and washed three times with 1× PBS. To reduce nonspecific staining, coverslips were washed in 0.1% Triton X‐100 in 1× PBS, for 5 min, followed by three washes in 1× PBS. Coverslips were counterstained for DNA with Bisbenzimide H 33342 stain (10 μg·mL^−1^) in 1× PBS 1 min and then washed three times in 1× PBS. Coverslips were mounted on microscope slides using the SlowFade^®^ Gold Antifade Reagent (Thermo Fisher Scientific), sealed, and stored at 4 °C until imaged. Imaging was performed in Virginia Commonwealth University Microscopy Facility maintained by the Department of Anatomy and Neurobiology (Virginia Commonwealth University, Richmond, VA, USA). Dual‐labeled microscope slides were imaged using the Zeiss LSM700 confocal laser scanning microscope (Carl Zeiss Microscopy, Thornwood, NY, USA) configured around an Axio Imager stand with four solid state lasers (405, 488, 555, and 639 nm) equipped with a 63×/1.4 NA Plan Achromat oil immersion objective lens. Multichannel images were collected sequentially to ensure no cross talk between channels, and the detector offset and gain settings were maintained for all of the images collected within a series. *Z*‐stack images were obtained at a frequency of 0.39 μm per optic slice. Quantitative analysis of colocalization was performed on confocal images using the colocalization function of the zeiss zen software (v. 8.0; Carl Zeiss Microscopy) or volocity program (v. 6.3; PerkinElmer Life Sciences, Waltham, MA, USA). Thresholds were set for each channel according to signal intensity and background noise. Colocalization coefficient (zen) or colocalization by intersecting volumes (volocity) as well as PCC were calculated.

### Immunoblot

Twenty microgram of protein from whole‐cell lysate or 15 μL of eluted proteins from immunoprecipitation were separated by Tris/glycine SDS/PAGE (8% α‐actinin, 10% tubulin or actin, 12% SIKE), and transferred to a nitrocellulose membrane. The membrane was blocked in either 5% milk or 5% BSA (rabbit α‐tubulin‐HRP) in TBS/Tween. The immunoblot was probed for SIKE using mouse anti‐FLAG‐HRP or mouse anti‐HA or rabbit anti‐SIKE or cytoskeletal protein using either mouse‐α‐GFP (BioLegend) or rabbit α‐tubulin‐HRP (Cell Signaling Technology, Danvers, MA, USA) at 4 °C overnight. The blot was then washed with TBS/Tween: 6 × 5 min (rabbit anti‐FLAG‐HRP) or 3 × 5 min (all other blots). For blots requiring a secondary antibody, goat anti‐mouse or goat anti‐rabbit IgG‐HRP (Southern Biotech, Birmingham, AL, USA), was incubated with blot for 1 h, and washed with TBS/Tween (4 × 10 min). See Table 2 for antibody dilutions. Membranes were developed with Pierce ECL Plus reagent (Thermo Fisher Scientific) as per manufacturer's protocol and documented on a Gel Doc XR+ gel documentation system (Bio‐Rad) or a c‐DiGit blot scanner (LI‐COR, Lincoln, NE, USA). For whole lysate immunoblots, the blots were stripped, blocked in 5% milk TBS/Tween, probed for actin using mouse anti‐actin‐HRP, washed with TBS/Tween (7 × 5 min), and developed as described above.

**Table 2:** Antibodies used in SIKE interaction assays

Cytoskeletal protein immunoprecipitation		
Mouse anti‐GFP	BioLegend, cat. #: MMS‐118P	7.5 μg
Rabbit anti‐mCherry	Thermo Fisher Sci, cat. #: PA5‐34974	7.5 μg
Immunoblots		
Rabbit anti‐SIKE	Sigma‐Aldrich, cat. #: HPA025726	1 : 300
Mouse anti‐FLAG‐HRP	Sigma‐Aldrich, cat. #: A8592	1 : 1000 (IP/IB)
Mouse anti‐FLAG‐HRP	Sigma‐Aldrich, cat. # A8592	1 : 10000 (lysate IB)
Mouse anti‐GFP	BioLegend, cat. #: MMS‐118P	1 : 5000
Mouse anti‐HA	BioLegend, cat. #: 16B12	1 : 1000 (IP/IB)
Mouse anti‐HA	BioLegend, cat. #: 16B12	1 : 1600 (lysate IB)
Goat anti‐rabbit IgG‐HRP	Southern Biotech, cat. #: 4030‐05	1 : 3000
Goat anti‐mouse IgG‐HRP	Southern Biotech, cat. #: 1031‐05	1 : 5000
Rabbit anti‐tubulin‐HRP	Cell Signaling Tech, cat. #: 11H10	1 : 1000
Mouse anti‐actin‐HRP	Sigma‐Aldrich, cat. #: A3854	1 : 10000 or 1 : 25000
Immunofluorescence		
Rabbit anti‐SIKE	Sigma‐Aldrich, cat. #: HPA024177	2 ng·μL^−1^
Mouse anti‐Rab5	Santa Cruz Biotech, cat. #:sc‐46692	1 : 200
Mouse anti‐Rab7	Santa Cruz Biotech, cat. #: sc‐271608	1 : 200
Mouse anti‐Rab11A	Santa Cruz Biotech, cat. #: sc‐166523	1 : 200
Mouse anti‐LAMP1	BioLegend, cat. # 328601	1 : 250
Mouse anti‐LC3	Santa Cruz Biotech, cat. #:sc‐271625	1 : 200
Mouse anti‐NPM1	Thermo Fisher Sci. cat. #: MA5‐17141	1 : 200
Mouse anti‐fibrillarin	Invitrogen, cat. # 480009	1 : 500
Mouse anti‐EDC4	Santa Cruz Biotech, cat. #: sc‐374211	1 : 200
Mouse anti‐PABP	Santa Cruz Biotech, cat. #:sc‐166381	1 : 200
Mouse anti‐S6 ribosomal protein	Cell Signaling Tech, cat. # 2317	1 : 25
Alexa Fluor 555 phalloidin	Thermo Fisher Sci, cat. # A34055	1 : 40
Mouse anti‐α‐actinin	Sigma‐Aldrich, cat. # A5044	1 : 300
Mouse anti‐α‐tubulin	Cell Signaling Tech, cat. #: 3873	1 : 4000
Mouse anti‐ezrin	Thermo Fisher Sci, cat. #: MA5‐13862	1 : 200
Mouse anti‐MLC2	Cell Signaling Tech., cat. #: 3675	1 : 200
Mouse anti‐FAK	Thermo Fisher Sci, cat. #: MA5‐15588	1 : 500
Mouse anti‐β‐catenin	Santa Cruz Biotech, cat. # sc‐7963	1 : 250
Goat anti‐rabbit Alexa Fluor 488	Cell Signaling Tech, cat. #: 4412	1 : 1000
Goat anti‐mouse Alexa Fluor 555	Thermo Fisher Sci, cat. #: A21422	1 : 1000

### Protein expression and purification

For pET15b (N‐terminal 6xHis tag) and pET26b (C‐terminal 6xHis tag) SIKE construct expression, vector was transformed into chemically competent BL21‐CodonPlus (DE3)‐RIPL (Agilent, Santa Clara, CA, USA) following the manufacturer's protocol. A single colony was used to inoculate an overnight culture of Luria broth plus 100 μg·mL^−1^ ampicillin. The overnight culture was subcultured 1 : 100 into 1 L LB/amp flasks grown at 37 °C until the cell density reached an A600 ≈ 0.6. Protein expression was induced with 1 mm isopropyl‐β‐d‐galactopyranoside and allowed to grow overnight at 20 °C. Cells were harvested by centrifugation at 7000 ***g***. Cells were resuspended in 50 mm NaH_2_PO_4_, pH 8, 300 mm NaCl, 1 mm 2‐mercaptoethanol, 0.5 mg·mL^−1^ lysozyme, and 1× complete EDTA‐free protease inhibitor and sonicated to lyse cells. Insoluble material was pelleted by centrifugation (14 000 ***g***, 30 min) and the pellet solubilized in guanidine hydrochloride (GudHCl) buffer (6 m guanidine hydrochloride, 50 mm NaH_2_PO_4_ (pH 8.0), 300 mm NaCl and 1 mm 2‐mercaptoethanol). Resuspended insoluble material was clarified by centrifugation at 14 000 ***g***, 30 min. The supernatant was mixed with 1.5 mL TALON resin (Clontech Laboratories, Inc., Mountain View, CA, USA), pre‐equilibrated in GudHCl buffer. The lysate resin mixture was loaded into a column (Bio‐Rad) and washed by gravity with 50 column volumes (CV) of GudHCl buffer. Bound protein was refolded on the column using a 133 CV reverse gradient of GudHCl buffer to 50 mm NaH_2_PO_4_ (pH 8.0), 300 mm NaCl and 1 mm 2‐mercaptoethanol (Buffer 1) and eluted with 5 CV of Buffer 1 plus 500 mm imidazole. For pET26b, an additional 50 CV wash Buffer 1 + 50 mm Imidazole was incorporated prior to elution to remove a SIKE fragment (10–14 kDa). Protein purity was assessed by SDS/PAGE analysis and protein quantified by the Bradford method (Bio‐Rad).

### *In vitro* precipitation

SIKE (8 μm final concentration) and cytoskeletal proteins, tubulin and α‐actinin (Cytoskeleton, Inc., Denver, CO, USA), or control protein, lysozyme, were incubated at molar ratios of 1 : 0.5, 1 : 1, or 1 : 1.4 in 50 mm NaH_2_PO_4_, pH 8, and 0.3 m NaCl (EQ buffer) at 4 °C. Reactions were added to Ni‐NTA agarose (50 μL, pre‐equilibrated in EQ buffer) and incubated at RT with constant agitation. Beads were pelleted by centrifugation (100 ***g***, 3 min) and supernatant (Unbound (UB) sample) was retained for analysis. Beads were washed three times with 10 CV EQ buffer + 50 mm imidazole. 3 CV EQ buffer + 250 mm imidazole was added to beads, immediately transferred to new tube, and incubated for 10 min to release bound sample. Beads were pelleted by centrifugation (100 ***g***, 3 min) and supernatant (bound (B) sample) was retained for analysis. Samples were separated by SDS/PAGE and stained with Coomassie blue or silver stain.

## Author contributions

HAS performed RcIP studies, analyzed the data, and wrote the manuscript; ELD and JBK performed RcIP studies and analyzed the data; FAS and TMM performed IVP studies and analyzed the data; KFL performed IFA studies and analyzed the data; KAW and SMF performed scratch assays and analyses; JDM performed co‐immunoprecipitations for MS/MS experiments; and JKB planned experiments, performed transfections for MS experiments, analyzed the data, and wrote the manuscript.
